# Fluid shear stress modulation of hepatocyte-like cell function

**DOI:** 10.1007/s00204-016-1689-8

**Published:** 2016-03-15

**Authors:** Hassan Rashidi, Sharmin Alhaque, Dagmara Szkolnicka, Oliver Flint, David C. Hay

**Affiliations:** MRC Centre for Regenerative Medicine, University of Edinburgh, Edinburgh, EH16 4UU UK

**Keywords:** Fluid shear stress, Hepatocyte-like cell, Embryonic stem cell, Cytochrome P450 Metabolism, Albumin secretion, Alpha-fetoprotein secretion

## Abstract

Freshly isolated human adult hepatocytes are considered to be the gold standard tool for in vitro studies. However, primary hepatocyte scarcity, cell cycle arrest and the rapid loss of cell phenotype limit their widespread deployment. Human embryonic stem cells and induced pluripotent stem cells provide renewable sources of hepatocyte-like cells (HLCs). Despite the use of various differentiation methodologies, HLCs like primary human hepatocytes exhibit unstable phenotype in culture. It has been shown that the functional capacity can be improved by adding back elements of human physiology, such as cell co-culture or through the use of natural and/or synthetic surfaces. In this study, the effect of fluid shear stress on HLC performance was investigated. We studied two important liver functions, cytochrome P450 drug metabolism and serum protein secretion, in static cultures and those exposed to fluid shear stress. Our study demonstrates that fluid shear stress improved Cyp1A2 activity by approximately fivefold. This was paralleled by an approximate ninefold increase in sensitivity to a drug, primarily metabolised by Cyp2D6. In addition to metabolic capacity, fluid shear stress also improved hepatocyte phenotype with an approximate fourfold reduction in the secretion of a foetal marker, alpha-fetoprotein. We believe these studies highlight the importance of introducing physiologic cues in cell-based models to improve somatic cell phenotype.

## Introduction

Static and two-dimensional (2D) culture systems have been used extensively to study human biology. Although those systems are facile and cost-effective to use, they lack the complexity of the three-dimensional (3D) tissue microenvironment. As a result, those systems do not accurately model tissue physiology and can generate inaccurate datasets. It is a well-known phenomenon that cells respond to physical and chemical stimuli provided by the tissue niche (Rashidi et al. 2014). Therefore, the add-back of human physiology to cell based models is of the utmost importance. This will likely lead to an improvement in cell phenotype and more informative biological readouts from those systems (Godoy et al. 2015).

Fluid shear stress is one mechanical stimulus that is absent in static culture systems. The role of fluid transport is fundamental for organogenesis (Freund et al. 2012), cell signalling (Mammoto and Ingber 2010) and normal patterns of organ function (Hahn and Schwartz 2009; Hildebrandt et al. 2011). Living cells possess the ability to sense mechanical forces and transduce those into biological responses (Bao and Suresh 2003; Freund et al. 2012). The mechanisms that modulate cell behaviour by fluid shear stress is diverse, but is mediated primarily through cell surface receptors (Tzima et al. 2001), cell adhesion molecules (Tzima et al. 2005) and heterodimeric G proteins (White and Frangos 2007).

The effects of fluid shear stress have also been studied in primary hepatocytes and transformed hepatocyte cell lines. In these studies, fluid shear stress has been shown to modulate cell viability (Park et al. 2008; Tilles et al. 2001) and significantly alter hepatocyte cytochrome P450 gene expression (Mufti et al. 1995; Mufti and Shuler 1996; Roy et al. 2001; Shvartsman et al. 2009; Vinci et al. 2011). In this study, the effect of fluid shear stress on human embryonic stem cell (hESC) derived HLC function was evaluated.

## Materials and methods

### Cell culture

hESCs (H9) were cultured as previously described (Szkolnicka et al. 2013, 2014a). Human ESCs were plated onto Matrigel^®^-coated (Corning^®^) Thermanox™ coverslips (Nunc). Monolayer differentiation was initiated at 40 % confluence by replacing serum-free medium mTESR1 (STEMCELL Technologies) with endoderm differentiation medium: RPMI 1640 containing 1 × B27 (Life Technologies), 100 ng/ml activin A (PeproTech) and 50 ng/ml Wnt3a (R&D Systems). The medium was changed every 24 h for 72 h. On day 4, endoderm differentiation medium was replaced with hepatoblast differentiation medium, and this was renewed every second day for a further 5 days. The medium consisted of knockout (KO)-DMEM (Life Technologies), serum replacement (Life Technologies), 0.5 % Glutamax (Life Technologies), 1 % non-essential amino acids (Life Technologies), 0.2 % b-mercaptoethanol (Life Technologies) and 1 % DMSO (Sigma). On day 9, differentiating cells were cultured in the hepatocyte maturation medium HepatoZYME (Life Technologies) containing 1 % Glutamax (Life Technologies), supplemented with 10 ng/ml hepatocyte growth factor (PeproTech) and 20 ng/ml oncostatin M (PeproTech) as described previously (Szkolnicka et al. 2014a). To differentiate HLCs in 3D, a single cell suspension of H9 hESCs was prepared as previously described (Szkolnicka et al. 2014a). H9s were re-suspended in mTESR1 medium containing ROCK inhibitor (Merck) at concentration of 1.9 × 10^5^ cells/ml. To form spheroids, 40 µl of the cell suspension was transferred into Perfecta3D^®^ plates (Biomatrix, USA) using a multichannel pipette. The following day, 3D cell cultures were transferred into Corning^®^ Costar^®^ Ultra-Low attachment multiwell plates and differentiated as previously described (Szkolnicka et al. 2014a). Spheroids were transferred at day 14 into a 24-well plate containing Matrigel-coated Thermanox™ (Nunc) coverslips and allowed to adhere prior to exposure to fluid shear stress.

### Quasi-vivo^®^ system set-up

Following 18 days of differentiation, HLCs grown on Thermanox™ coverslips were transferred into serially connected chambers of the Quasi-Vivo^®^ system (Kirkstall Limited, UK). The fluid shear stress system was transferred into a humidified 37 C, 5 % CO2 incubator, and fluid shear stress was applied at 2.9 × 10^−5^ and 4.7 × 10^−5^ dynes/cm^2^ for 18 h. Following fluid shear stress, cell populations were transferred to a 24-well plate for biochemical analysis.

### Cytochrome P450 assays

Eighteen hours post-fluid shear stress, Cyp1A2 metabolic activity was measured using pGlo technology (Promega) and carried out according to the manufacturer’s instructions (Cameron et al. 2015). Cyp1A2 activity was expressed as relative light units (RLUs) per millilitre of medium per milligram of protein (BCA assay, Pierce) per 5 h. Levels of significance were measured by Student’s *t* test. The experiments are representative of three biological replicates.

### Cell viability assays

Eighteen hours post-fluid shear stress, cell viability was measured using Cell Titer Glo (Promega) and carried out according to the manufacturer’s instructions (Szkolnicka et al. 2014b). ATP levels were expressed as relative light units (RLUs) per millilitre of cell culture medium. Levels of significance were measured by Student’s *t* test. The experiments are representative of three biological replicates.

### Albumin and alpha-fetoprotein ELISA

Eighteen hours post-fluid shear stress, HLC alpha-fetoprotein and albumin secretion were measured using commercially available ELISA kits (Alpha Diagnostic International). Protein secretion was expressed as microgram of protein per millilitre of medium per milligram of protein per 24 h. Levels of significance were measured by Student’s *t* test. The experiments are representative of three biological replicates.

## Results

**Fig. 1:**
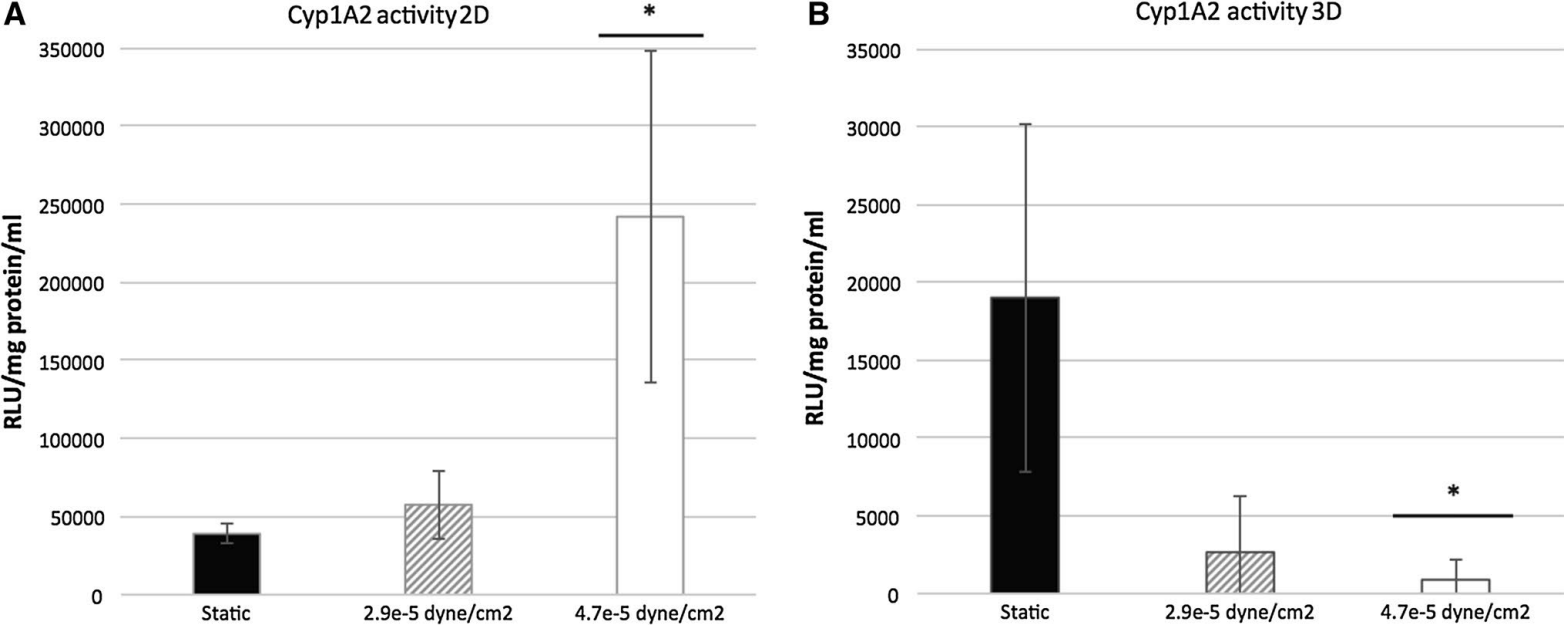
**a** Cyp1A2 metabolic activity of monolayer hepatocyte-like cells (HLCs) under static conditions (*black columns*) and following fluid shear stress (FSS) of 2.9 × 10^−5^ dynes/cm^2^ (*shaded columns*) and 4.7 × 10^−5^ dynes/cm^2^ (*white columns*). Data are presented as mean of three independent experiments. *Error bars* represent the standard deviation (SD). *p < 0.05, **p < 0.01; two-tailed *t* test analysis. **b** Cyp1A2 metabolic activity of 3D HLCs under static conditions (*black columns*) and following FSS of 2.9 × 10^−5^ dynes/cm^2^ (*shaded columns*) and 4.7 × 10^−5^ dynes/cm^2^ (*white columns*). Data are presented as mean of three independent experiments. *Error bars* represent SD. *p < 0.05, **p < 0.01; two-tailed *t* test analysis

**Fig. 2:**
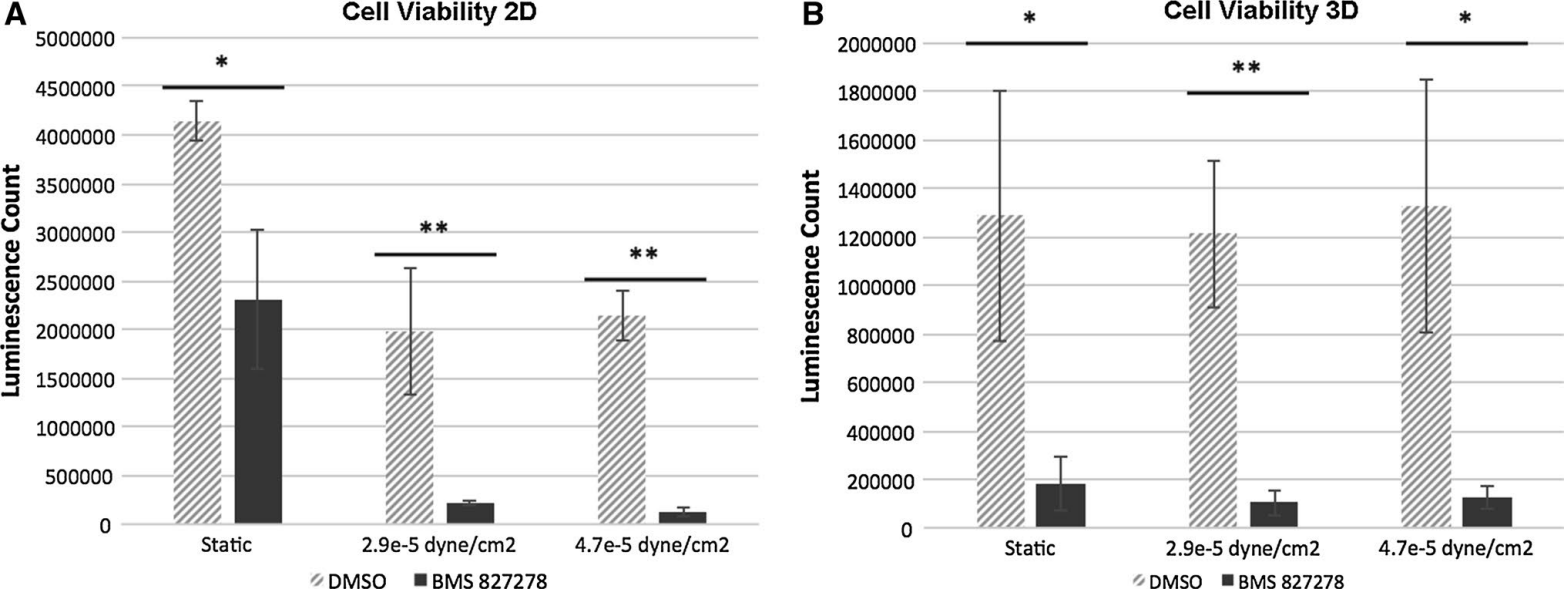
**a** Cell viability assay of monolayer cultured HLCs following exposure to DMSO and BMS 827278 under static conditions or following exposure to fluid shear stress (FSS). Data are presented as mean of three independent experiments. *Error bars* represent the standard deviation (SD). *p < 0.05, **p < 0.01; two-tailed *t* test analysis. **b** Cell viability assay of 3D cultured HLCs following exposure to DMSO and BMS 827278 under static conditions or following exposure to FSS. Data are presented as mean of three independent experiments. *Error bars* represent SD. *p < 0.05, **p < 0.01; two-tailed *t* test analysis

The effect of fluid shear stress (FSS) on stem cell-derived HLC metabolic activity was studied using the Quasi-Vivo^®^ system (Kirkstall Limited, UK). HLCs were exposed to FSS, ranging from 2.9 × 10^−5^ to 4.7 × 10^−5^ dyne/cm^2^. After 18 h FSS, HLCs were transferred into a 24-well plate for further analysis. Cytochrome P450 (Cyp) metabolic activities of two enzymes, Cyp1A2 and Cyp2D6, were measured to evaluate the effect of FSS on HLC phenotype. Cyp1A2 activity was significantly increased fivefold over controls in 2D cultures at 4.7 × 10^−5^ dyne/cm^2^ (Fig. 1a). To assess Cyp2D6 activity, a compound (BMS 827278) which requires Cyp2D6 metabolic activity to convert it to a toxic end point was employed. Post-exposure to BMS 827278, cell viability was measured and compared to the DMSO vehicle control. As expected, microscopic and quantitative ATP analysis revealed that BMS 827278 had a detrimental effect on the viability of HLCs (Medine et al. 2013; Villarin et al. 2015). Notably, HLC viability was significantly decreased by a further fivefold and ninefold in response to FSS at 2.9 × 10^−5^ and 4.7 × 10^−5^ dyne/cm^2^, respectively (Fig. 2a). This demonstrates that FSS was an important physiological stimulus for metabolic activity in human hepatocyte models derived from pluripotent stem cells.

**Fig. 3:**
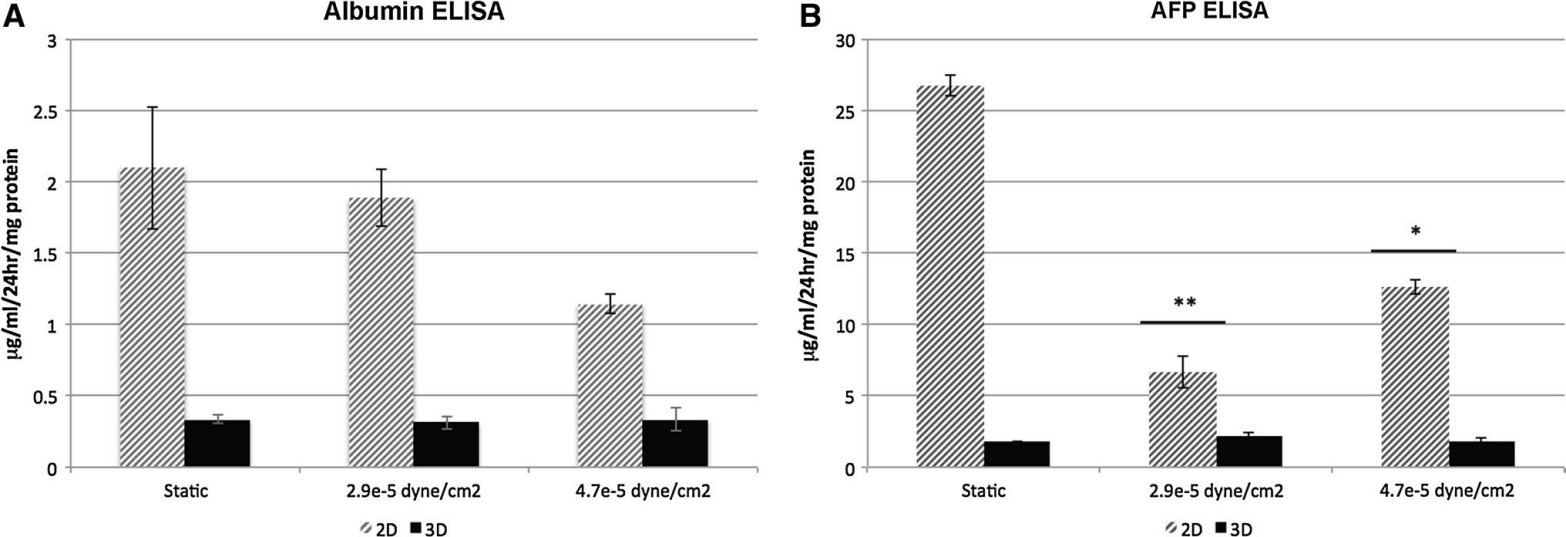
**a** HLC albumin production was measured by ELISA under static conditions or following exposure to fluid shear stress (FSS). Albumin secretion is expressed as micrograms of albumin (ALB) ml^−1^ per 24 h per mg protein. Data are presented as mean of three independent experiments. *Error bars* represent the standard deviation (SD). *p < 0.05, **p < 0.01; two-tailed *t* test analysis. **b** HLC AFP production was measured by ELISA under static conditions or under FSS. Alpha-fetoprotein (AFP) secretion is expressed as micrograms of AFP ml^−1^ per 24 h per mg protein. Data are presented as mean of three independent experiments. *Error bars* represent SD. *p < 0.05, **p < 0.01; two-tailed *t* test analysis

To examine the effect of FSS on 3D-cultured HLCs, hepatic spheroids were seeded on Matrigel-coated Thermanox™ coverslips prior to transfer into the Quasi-Vivo^®^ system. Anchored spheroids were then exposed to FSS of 2.9 × 10^−5^ and 4.7 × 10^−5^ dyne/cm^2^ for 18 h. Contrary to 2D-cultured HLCs, metabolic activity of Cyp1A2 decreased significantly following exposure to 4.7 × 10^−5^ dyne/cm^2^ by approximately twentyfold (Fig. 1b). Similar to 2D-cultured HLCs, 3D spheroids showed sensitivity to BMS 827278 which was marginally enhanced following FSS. The 3D spheroids cultured under static condition showed over a threefold increase in sensitivity to BMS 827278 in comparison with 2D cultures. This suggests that 3D cultures displayed an improved basal level of Cyp2D6 activity (Fig. 2b). In addition to metabolic activity, we also measured serum protein production under static conditions and flow. The production of albumin remained constant in 2D and 3D models under static conditions and in response to FSS (Fig. 3a). In contrast, the production of alpha-fetoprotein (AFP) was significantly reduced in 2D, but not 3D HLCs following exposure to FSS (Fig. 3b).

## Discussion

The liver is a highly vascular organ, processing ~25–30 % of the total blood volume at any given time (Bradley et al. 1945). Arterial and venous blood enters the liver lobes, flowing through the sinusoids towards the central vein (Ebrahimkhani et al. 2014). Sinusoids commonly have diameters ranging from 7 µm in the periportal to 15 µm in pericentral regions (Vollmar and Menger 2009) and experience fluid shear stresses between 0.1–0.5 dyne/cm^2^. This is lower than fluid shear stresses observed in other capillary systems, typically around 15 dyne/cm^2^ (Koutsiaris et al. 2007). While it is difficult to gauge the exact level of shear stress experienced by hepatocytes (LeCluyse et al. 2012), it has been estimated to be several orders of magnitude lower than the level of sinusoidal shear stress.

Despite the evidence supporting the importance of FSS in cell biology, few studies have addressed this in the hepatocyte. In pioneering work, Mufti and colleagues showed transient increase in AhR-driven Cyp1A1 expression and function following exposure to FSS (Mufti et al. 1995). A later study suggested a potential role of arachidonic acid in the induction of Cyp1A1 in HepG2 cells under hydrodynamic stimulation (Mufti and Shuler 1996). Along similar lines, the induction of Cyp1A1 activity has also been reported in rat hepatocytes (Gebhardt et al. 1996; Roy et al. 2001). More recently, genes involved in human drug metabolism, including CYP1A1, 1A2, 2B6, 2C9 and 3A4, were shown to be positively regulated by flow (Vinci et al. 2011). Our data provide further evidence to support the importance of FSS on HLC metabolic function and serum protein secretion (Figs. 1, 2, 3).

## Conclusion

In conclusion, a more active and predictive hepatocyte model was created after exposure to fluid shear stress. We believe that this demonstrates the importance of adding back human physiology to improve somatic cell-phenotype in vitro.
